# Meibomian Gland Dysfunction and Dropout in Diabetic Patients with Non-Proliferative Diabetic Retinopathy

**DOI:** 10.3390/bioengineering11090907

**Published:** 2024-09-10

**Authors:** Karim Mohamed-Noriega, Carla Sofía González-Arocha, Fernando Morales-Wong, Braulio Hernán Velasco-Sepúlveda, Jonathan Octavio Rodríguez-Cuevas, Gerardo Esteban Cepeda-Ortegón, Sergio Antonio Corral-Benavides, José Francisco Martínez-Delgado, Jibran Mohamed-Noriega, Marissa L. Fernández-De-Luna, Jesús Mohamed-Hamsho

**Affiliations:** Ophthalmology Department, University Hospital, Faculty of Medicine, Autonomous University of Nuevo Leon (UANL), Monterrey 64460, Mexico; carla.gonzalezarc@uanl.edu.mx (C.S.G.-A.); fernando.moraleswn@uanl.edu.mx (F.M.-W.); braulio.velascosplv@uanl.edu.mx (B.H.V.-S.); dr.jrdz2017@gmail.com (J.O.R.-C.); gerardo.cepedaor@uanl.edu.mx (G.E.C.-O.); sergio.corralbn@uanl.edu.mx (S.A.C.-B.); jose.martinezdld@uanl.edu.mx (J.F.M.-D.); jibran.mohamednrg@uanl.edu.mx (J.M.-N.); marissa.fernandezd@uanl.edu.mx (M.L.F.-D.-L.); jesus.mohamedhms@uanl.edu.mx (J.M.-H.)

**Keywords:** meibomian gland dropout, meibography, meibomian gland dysfunction, dry eye disease, diabetes, diabetic retinopathy

## Abstract

This study aims to compare meibomian gland (MG) dropout and MG dysfunction (MGD) between patients with diabetes mellitus (DM) with moderate–severe non-proliferative diabetic retinopathy (NPDR) and patients with no diabetes (NDM). This prospective, transversal, age, and gender-matched case–control study included 98 DM and 106 NDM eyes. Dry eye disease (DED) and MGD evaluations were performed, including meibography (Keratograph 5M^®^). The objective MG dropout percentage was obtained by analyzing meibography images with ImageJ software (v. 1.52o, National Institutes of Health, Bethesda, MD, USA) and was subsequently graded with Arita’s meiboscore. The DM duration was 18 ± 9 years. The mean meiboscore (3.8 ± 0.8 vs. 3.4 ± 1.0, *p* = 0.001), meiboscore severity (*p* = 0.016), and MG dropout (45.1 ± 0.1% vs. 39.0 ± 0.4%, *p* < 0.001) were greater in DM than in NDM. All patients showed MG dropout (meiboscore > 1). Lower eyelids showed greater MG dropout in both groups. A correlation with age (r = 0.178, *p* = 0.014) and no correlations with DM duration or gender (*p* > 0.005) were observed. Patients with diabetes showed greater corneal staining (1.7 ± 1.3 vs. 0.9 ± 1.1; *p* < 0.001), reduced corneal sensitivity (5.4 ± 1.1 vs. 5.9 ± 0.4; *p* < 0.001), lower MG expressibility (3. 9 ± 1.6 vs. 4.4 ± 2.1; *p* = 0.017), and worse meibum quality (1.9 ± 0.8 vs. 1.7 ± 0.5; *p* = 0.019). Tear breakup time, osmolarity, MMP-9, Schirmer, and the Ocular Surface Disease Index showed no significant differences. In conclusion, patients with DM with NPDR have greater MG dropout and meiboscore, as well as more severe MGD and DED parameters than persons with NDM.

## 1. Introduction

TFOS DEWS II defines dry eye disease (DED) as a multifactorial disease of the tears and ocular surface that results in symptoms of discomfort, visual disturbance, and tear film instability with potential damage to the ocular surface. It is accompanied by an increased osmolarity of the tear film and inflammation of the ocular surface [1]. Evaporative DED is the most frequent DED subtype, and meibomian gland dysfunction (MGD) is a major cause of dry eyes [1,2,3]. MGD is defined as a chronic, diffuse abnormality of the meibomian glands (MG), commonly characterized by terminal duct obstruction and/or qualitative/quantitative changes in glandular secretion [2,4]. The estimated global prevalence of diabetes mellitus (DM) is predicted to rise to 642 million in 2040 [5]. Patients with DM are at an increased risk of developing DED, having more frequent and more severe DED and MGD than patients with no diabetes [1,2,3,6,7,8]. The impact that DM may have on DED and MGD function is due to both microvascular and neuropathic changes in the tear gland, meibomian glands, cornea, and conjunctiva. This is probably due to the autonomic dysfunction and peripheral neuropathy that patients with DM have [2,6,7,8]. The quality and quantity of meibum are affected by insulin resistance and deficiency; hyperglycemia has a detrimental effect on sterols and lipid receptors in the glands. All this subsequently induces MG obstruction, reduced MG expressivity, worse meibum, and ultimately a deficient and altered tear lipid layer [1,2,3,6,7,8].

Meibography allows for a direct visualization of MG and, therefore, an objective identification of MG damage, observed as MG dropout (loss of MG or reduction in its size secondary to shortening and atrophy of the main ducts), tortuosity, and thinning [9,10]. MG dropout grading is usually performed subjectively [11]. However, it is possible and desirable to perform objective MG image analysis with ImageJ software (National Institutes of Health, Bethesda, MD, USA) to obtain a more precise percentage of MG dropout and, as a consequence, more reliable grading [9]. Briefly, ImageJ software can calculate the area and pixel value of user-defined selections in any given image. The specific areas of interest are chosen with selection tools, which allow freehand, multipoint, or line selection. First, the total MG area is manually selected, and then the area of MG loss is selected, and from that, the percentage of the MG area lost can be calculated.

There are scarce papers about MGD and diabetes that include an assessment of MG dropout with meibography. Only one published study performed objective and quantitative analysis of MG dropout [12]; most other studies only performed subjective grading and analysis (Table 1). There is a wide range of diabetes duration in the available manuscripts, and they do not mention whether patients with diabetic retinopathy were included (Table 1). Therefore, this study aims to assess whether the percentages of MG dropout, meiboscore grade, MGD, and DED parameters in patients with DM with non-proliferative diabetic retinopathy (NDPR) are more severe than in patients with no diabetes, as evaluated with objective and quantitative meibography image analysis methods.

## 2. Materials and Methods

This was a prospective, transversal case–control study of patients with DM with NPDR listed for cataract surgery. Patients were recruited between January 2016 and July 2018 at the ophthalmology department of the University Hospital of the Autonomous University of Nuevo León (UANL), a tertiary care university hospital in Monterrey, Mexico. This study received institutional ethics committee approval (ID: OF15-002) and was conducted in accordance with good clinical practices and the Declaration of Helsinki. All patients read and provided written informed consent to participate.

Patients listed for cataract surgery, older than 18 years and of both genders, were included. The group with diabetes included patients with moderate–severe NPDR. Diagnosis of DM was performed by an internal medicine physician following the American Diabetes Association diagnostic criteria [16]. Moderate-severe NPDR diagnosis was performed following the International Clinical Diabetic Retinopathy Disease Severity Scale [17]. The group with no diabetes, which served as control, included patients listed for cataract surgery without diabetes. These patients underwent blood tests to measure fasting glucose levels and an evaluation by an internal medicine physician to exclude the presence of DM following the American Diabetes Association diagnostic criteria [16]. Patients with rheumatic disease, Sjögren syndrome, ocular rosacea, graft versus host disease, pterygium, uveitis, ocular inflammation, previous ocular surgery, contact lens use, and glaucoma eye drops were excluded.

Before cataract surgery, all patients underwent a complete ophthalmic and dry eye evaluation, including medical history, demographics, ocular and systemic diseases, and medications. The subjects were instructed not to apply eye drops for at least 2 h before clinical evaluations [18,19,20]. Ophthalmic and dry eye evaluations were performed in both eyes as follows: Ocular Surface Disease Index (OSDI) questionnaire (Allergan, Inc., Irvine, CA, USA) [21], tear osmolarity with TearLab® (TearLab Corporation, San Diego, CA, USA), and qualitative positive tear matrix metalloproteinase-9 (MMP-9) with InflammaDry® (Quidel corporation, CA, USA) designed to detect abnormally elevated MMP-9 levels (>40 ng/mL). Tear film stability was assessed with fluorescein tear film breakup time (TBUT) at a slit lamp with a cobalt blue filter, described as follows: Fluorescein was instilled into the temporal inferior fornix with a fluorescein sodium strip (BioGlo^TM^, Hub Pharmaceuticals, CA, USA). We waited 20 s for fluoresceine to distribute in the tear film; then the patient was asked to blink 3 times and keep both eyes open. TBUT was defined as the duration in seconds required for the first area of the tear film to break up. Immediately after TBUT, the cornea staining with fluorescein was recorded with Oxford and NEI grading. Schirmer’s test without and with anesthesia (topical tetracaine) was performed by placing a sterile strip (TearFlo ^TM^ 35 mm strip, Hub Pharmaceuticals, CA, USA) into the inferior fornix, at the junction of the middle and lateral third of the lower eyelid margin for 5 min with both eyes closed. Central corneal sensitivity was measured using a Cochet–Bonnet esthesiometer (Luneau Ophthalmology, Paris, France). Eyelid margin evaluation included MG expressibility (a stable pressure was applied to the central third of the lower eyelid, and the number of expressible glands was recorded from 0 to 8, where 0 means no expressible glands and 8 means all glands are expressible) [4,15] and meibum quality (0: normal, clear, may have few particles; 1: opaque with normal viscosity; 2: opaque with increased viscosity; and 3: severe thickening (toothpaste like) [4], lid margin, and Marx’s line (1: normal; 2: thickened; 3: anteriorized; 4: posteriorized).

**Figure 1:**
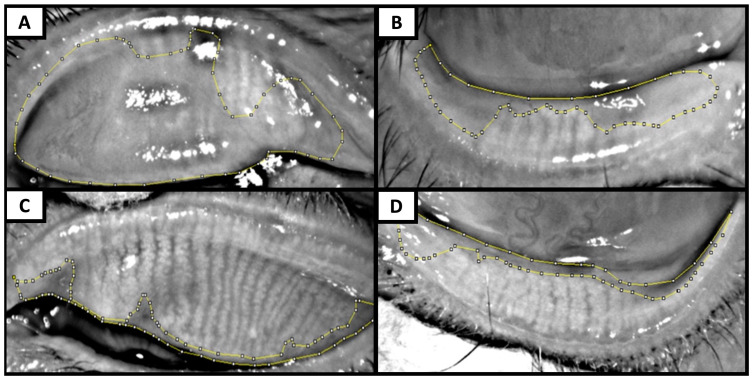
Representative meibography images show meibomian gland dropout assessment with ImageJ and Arita’s meiboscore grading score. (**A**,**B**) Superior and inferior eyelid of a patient with diabetes with non-proliferative diabetic retinopathy with meibomian gland (MG) dropouts of 72% (Arita’s grade 3) and 44% (Arita’s grade 2), respectively. (**C**,**D**) Superior and inferior eyelid of a patient with no diabetes with MG dropouts of 12% (Arita’s grade 1) and 18% (Arita’s grade 1), respectively.

Meibography was performed with Keratograph 5M^®^ by everting the upper and lower eyelids to expose the inner conjunctiva and the embedded meibomian glands. Care was taken to obtain the images that had the best possible quality, were well focused, and had as few reflections and shadows as possible [10]. A single-blinded investigator analyzed the meibography images using the image editing ImageJ software (v. 1.52o, National Institutes of Health, Bethesda, MD, USA) to perform a quantitative assessment of the percentage of MG dropout [9,11,15,16,17,21,22]. First, the tarsal area of each tarsus was selected in the meibography; then the MG loss area was selected. The percentage of MG dropout for each tarsus was calculated with this formula: MG loss area/total tarsal area (Figure 1). With the percentage of MG dropout, meibography images were graded according to Arita’s meiboscore grading scale [11,23]. In Arita’s meiboscore grading scale, each eyelid is scored from 0 to 3 (0: 0%; 1: <33%; 2: 33–66%; 3: >66% of MG dropout), and the sum of the upper and lower eyelid scores is the total meiboscore, which ranges from 0 to 6 for each eye [11,23]. MG dropout was defined as a meiboscore greater than 1 [2,6,24]. Representative images of MG dropout assessment and Arita’s meiboscore classification are presented in Figure 1.

The sample size was calculated with G*Power [25] based on the meiboscore difference between groups of 0.7 ± 1.4 SD to achieve an effect size (d) of 0.5, a significance level (α error prob) of 0.05, and a power (1- β err prob) of 0.9 in a two-tailed test, yielding a sample size of 86 eyes each group [15]. Data are presented as means and standard deviations (SD) for continuous variables and as frequencies and percentages for categorical variables. The distribution of data was analyzed with the Kolmogorov–Smirnov test. Pearson’s X2 test was used to compare categorical data, and a two-tailed independent *t*-test was used to compare continuous variables. Correlations were performed with Pearson’s coefficient test. Significance was considered when *p* ≤ 0.05. Statistical analysis was performed using IBM SPSS Statistics for Windows, Version 20.0 (Armonk, NY, USA: IBM Corp).

## 3. Results

Ninety-eight eyes of 49 patients with DM with NPDR (24 men and 25 women) and 106 eyes of 53 NDM control patients (26 men and 27 women) were included. The mean ± SD age was 67.1 ± 10.0 and 66.7 ± 9.0 years for patients with NPDR and patients with no diabetes, respectively (*p* = 0.982). The mean ± SD (min–max) diabetes duration in patients with DM and NPDR was 17.88 ± 8.48 (3–31) years (Table 2). Patients with DM had significantly greater cornea staining scores, reduced corneal sensitivity, lower MG expressibility, worse meibum quality, and a more advanced Marx’s line than patients with no diabetes (*p* < 0.05). No significant differences were observed between study groups in OSDI, osmolarity, MMP-9, Schirmer’s, and TBUT tests (Table 2).

**Figure 2:**
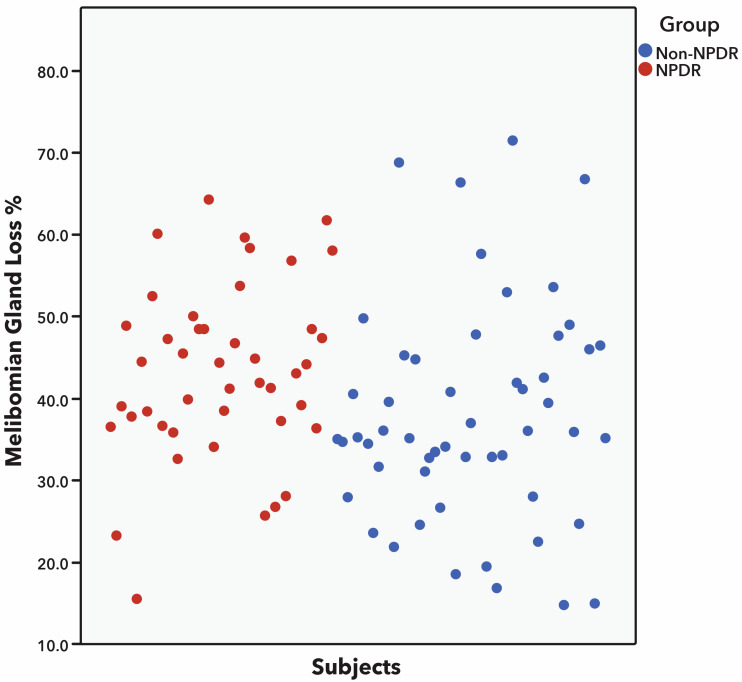
Scatter dot plot showing greater percentage of meibomian gland (MG) dropout in patients with diabetes with non-proliferative diabetic retinopathy (NPDR) than in patients with no diabetes. Only few patients with NPDR had less than 33% of MG dropout (equivalent to meiboscore grade 1), whereas significantly more patients in the group with no diabetes had MG dropout grade 1 (*p* < 0.001). Statistical analysis was performed with Student’s *t*-test.

**Figure 3:**
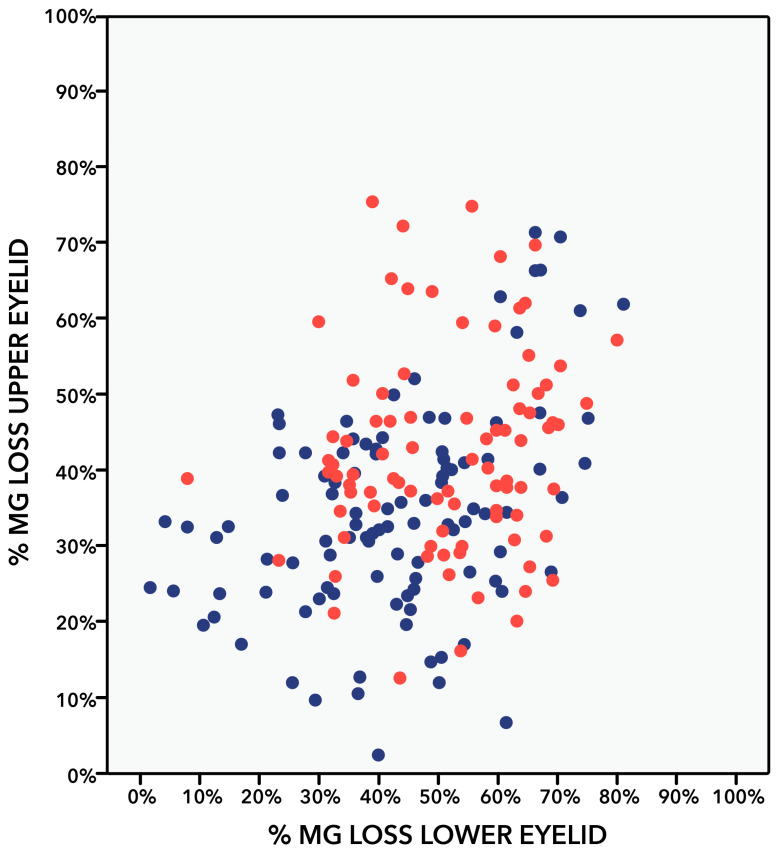
Scatter dot plot comparing the percentages of meibomian gland (MG) dropout in superior and inferior eyelid of patients with diabetes with non-proliferative diabetic retinopathy (NPDR) (red) and patients with no diabetes (blue). Pearson correlation test. Significance at *p* < 0.05. Patients with no diabetes: r = 0.429 (*p* < 0.001). Patients with diabetes with NPDR: r = 0.137 (*p* = 0.201).

Patients with DM and NPDR had a significantly greater percentage of MG dropout and greater meiboscore than patients with no diabetes when analyzing superior eyelid, inferior eyelid, and both eyelids (Table 3). The mean meiboscore was significantly greater in patients with DM with NPDR than in patients with no diabetes (3.8 ± 0.8 vs. 3.4 ± 1.0; *p* = 0.002) with a mean difference (confidence interval, CI) between groups of −0.42 (−0.67 to −0.16) (*p* = 0.002). Similarly, the mean percentage of MG dropout was significantly greater in patients with DM with NPDR than in patients with no diabetes (45.1 ± 10.5 vs. 39.0 ± 13.2; *p* < 0.001) with a mean difference (CI) between groups of −7.78 (−11.21 to −4.35) (*p* < 0.001) (Table 3 and Figure 2). When comparing the upper and lower eyelids with each other, it was found that the lower eyelid had higher MG dropout and Arita’s grading score than the upper eyelid in patients with DM with NPDR and in patients with no diabetes (Table 3 and Figure 3).

All patients in both groups showed some degree of MG dropout defined as a meiboscore greater than 1. However, the severity of the meiboscore was significantly greater in patients with DM with NPDR than in patients with no diabetes (*p* = 0.016). Most patients with diabetes with NPDR showed a meiboscore equal to or greater than 4. On the contrary, most patients with no diabetes had a meiboscore of 2–3 (Table 4 and Figure 2). Significant correlations were observed between the percentage of MG dropout and the evaluated dry eye parameters in patients with NPDR. Greater percentages of MG dropout were correlated with reduced TBUT (r = −0.295, *p* = 0.006), reduced MG expressibility (r = −0.428, *p* = 0.002), and more advanced Marx’s line (r = 0.371, *p* = 0.007). No significant correlations were observed in patients with no diabetes (Table 5). In patients with DM with NPDR, no gender differences were found in MG dropout (male: 45.8% vs. female: 47.1%, *p* = 0.580) and meiboscore (male: 3.8 vs. female: 3.9, *p* = 0.545). Similarly, in patients with no diabetes, no gender differences were found in MG dropout (male: 39.4% vs. female: 38.2%, *p* = 0.644) or meiboscore (male: 3.5 vs. female: 3.4, *p* = 0. 609). No statistically significant correlations were observed between DM duration and MG dropout (r = −0.186, *p* = 0.172) or meiboscore (r = −0.073, *p* = 0.599). On the contrary, age showed a weak but statistically significant correlation with MG dropout (r = 0.178, *p* = 0.014) and meiboscore (r = 0.257, *p* = 0.001), showing that, with older age, there is more MG dropout.

## 4. Discussion

The findings of this study on adult patients listed for cataract surgery showed that all had some degree of MG dropout. However, patients with diabetes with NPDR had a greater percentage of MG dropout and greater meiboscore than patients with no diabetes. In addition, patients with DM with NPDR had more severe DED and MGD and were documented to have more corneal staining, reduced corneal sensitivity, less MG expressibility, worse meibum quality, and more severe Marx’s line, compared with patients with no diabetes. These findings of more severe MG dropout and more severe meiboscore agree with other authors’ findings [6,12,13,14,15]. Table 1 shows published studies about MG dropout and meiboscore in patients with type 2 diabetes and compares them with the current study [6,12,13,14,15].

Our study and that of Yang Q. et al. are the only two studies that report the actual percentage of MG dropout. Yang Q. et al. found greater MG dropout in patients with DM with DED than in patients with no diabetes without DED (33.5 ± 8.2 vs. 16.5 ± 6.6, *p* = 0.001) [12]. This agrees with our study, which found greater MG dropout in patients with DM than in controls with no diabetes (45 ± 11 vs. 39 ± 13, *p* = 0.016). This agrees with this study’s finding of greater MG dropout in DM (45 ± 11 vs. 39 ± 13, *p* = 0.016); however, MG dropout was greater, maybe because of longer diabetes duration, or all patients had NPDR. Tao Yu et al. considered the presence of MG dropout as a meiboscore greater than 1; they found that 57.63% of patients in the DM group had MG dropout, while only 33% in the control group (*p* < 0.001) did. Some limitations of this study are that they did not mention the mean meiboscore nor the mean percentage of MG dropout, and they performed subjective meiboscore evaluation [6]. In the present study, all patients had some degree of MG dropout, and the majority of patients with DM with NPDR had a meiboscore of 3–5, compared with patients with no diabees, where the majority of them had a meiboscore of 2–4. Although all these studies agree that patients with DM have greater MG dropout than patients with no diabetes, there are differences in the magnitude of the reported MG dropout between studies. These differences between studies might be related to several possible causes, such as age, meibography analysis technique, duration of diabetes, and the presence of diabetic retinopathy. The objective analysis is expected to be more precise than the subjective one; and older age, longer duration of diabetes, and diabetic retinopathy are expected to induce greater MG dropout [14,15,23].

The meibography images were analyzed with ImageJ software to obtain a reliable objective quantitative percentage of MG dropout. By performing this measurement, it is possible to obtain more reliable meibography grading scores [2,10,14,23,26]. Pult H. et al. [9] reported a better intra-observer and inter-observer agreement when meibography images were evaluated by computerized grading with ImageJ, followed by the subjective grading scale [9,11]. This is a strength of the current study because this study and the study of Yang Q. et al. are the only two that performed objective analysis with ImageJ, while the previous studies on DM and MG dropout analyzed only the subjective grading of meiboscore (Table 1).

Great variability was observed in the percentage of MG dropout in patients with NPDR (mean: 45.1 ± 10.5, min: 16, max: 64) and in patients with no diabetes (mean: 39.0 ± 13.2, min: 15, max: 72) (Table 3 and Table 4 and Figure 2). This is in agreement with previous reports that show wide meiboscore ranges in patients with diabetes and patients with no diabetes [6,14,15]. This can be attributed to the widespread occurrence and multifactorial causes of DED and MGD, which can lead to more severe MGD and MG dropout. For instance, individuals who have undiagnosed DED and MGD before developing diabetes might experience more severe MGD and MG dropout. Additionally, those who have poor diabetes control over their lifetimes may experience greater deterioration in MG dropout [14,27,28,29].

When comparing some demographic characteristics of the current study with others previously published, we found that the patients in the current study have similar ages to those in other studies and have more years of diabetes duration, and it is the only one that mentions the presence of diabetic retinopathy, whereas the other studies failed to mention it. We considered that mild retinopathy could represent a very early stage of the disease, and moderate–severe NPDR would include a population with a slightly more homogeneous level of severity. Results by Yang Q. and Wu Huping et al. reported a positive correlation between DM duration and MG dropout [12,13,14]. The current study found no significant correlation between DM duration and MG dropout or meiboscore. An explanation for these results might be that patients had longer DM duration, and all had NDPR. Several studies involving patients with DM have reported that the prevalence and severity of DED are in part related to DM duration [15,30,31,32], particularly when it has over 10 years of evolution [33]. Maniaviat MR et al. [30] found that a longer DM duration was significantly associated with a higher prevalence of DED. They also found that patients with diabetic retinopathy had a significantly higher prevalence of DED than patients with diabetes without DR [30]. Most studies on the subject did not investigate the correlation between age and MG dropout. Wu Huping et al. and the current study had similar findings and agree that a positive correlation exists between age and MG dropout in patients with DM (Table 1) [13].

There are multiple risk factors for developing dry eye and MGD [29]. It is well known that diabetes is associated with the development of MGD; however, MGD is underdiagnosed [15,34]. Therefore, the presence of diabetes and NPDR could be considered risk factors for the development of ocular surface damage in the form of MG dropout, MGD, and dry eye disease. In this study, patients with moderate–severe NPDR were assessed with the International Clinical Diabetic Retinopathy Disease Severity Scale. NPDR is an objective finding in the ophthalmic evaluation. Meanwhile, DM duration might be a misleading sign of disease evolution especially when diabetes diagnosis was not performed early in the disease. Diabetes is often first diagnosed in eye clinics in an advanced stage once visible signs of diabetic retinopathy have appeared [28], so the presence of a homogeneous organ damage such as NPDR from type 2 diabetes could be a more reliable risk factor than the duration of diabetes. In patients with diabetes, it is important not to be guided only by disease duration, because, in countries where the screening strategies do not cover the whole population, patients frequently show organ damage at the time of diagnosis. The findings of the present study can help ophthalmologists who identify diabetic retinopathy to increase their awareness of possible concomitant MG dropout, MGD, and dry eye disease, which can have a significant impact on patients’ quality of life. It would be very interesting to evaluate the differences in MG dropout in the different degrees of diabetic retinopathy; however, this study focused only on moderate–severe NPDR, and future studies can focus on evaluating the changes in different degrees of diabetic retinopathy. Although this study was conducted in a population of patients listed for cataract surgery, it did not study the impact that cataracts could have on MG dropout. It would also be interesting to evaluate this in future studies, although it is more likely that age may be related to MG dropout than the cataract itself. It is well known that cataract surgery is associated with DED worsening, although perhaps only temporary. In this study, we did not evaluate longitudinally whether cataract surgery caused or deteriorated their DED, MGD, or MG dropout, nor did it evaluate the need for the use of artificial tears. It would be very interesting to evaluate in future studies the impact of cataract surgery in MGD and MG dropout in patients with diabetes or diabetic retinopathy.

In this study, patients with DM with NPDR and patients with no diabetes showed severe dry eye symptoms based on their OSDI scores, but no significant differences were observed between groups. This could be influenced by the blurred vision all patients presented with, since all patients were listed for cataract surgery, and by the fact that there is a high prevalence of dry eye symptoms in the general population [29]. The etiology and pathogenesis of dry eye in patients with diabetes are likely multifactorial due to both microvascular and neuropathic changes in the lacrimal gland, meibomian glands, cornea, and conjunctiva [28,35,36]. In the present study, patients with DM with NPDR had lower corneal sensitivity than patients with no diabetes. This is in agreement with the study by Saini JS et al. [35], who showed that patients with NPDR have lower corneal sensitivity compared with patients with no diabetes and patients with diabetes without retinopathy [35]. A long-term complication of diabetes is peripheral neuropathy [15,36], including damage to the corneal nerves (decreased sub-basal nerve density and increased dendritic cell density in the central cornea) [28], which leads to reduced corneal sensitivity [15,32,34,37]. The increased damage to the ocular surface in patients with NPDR observed as increased corneal staining can be attributed to multiple factors, such as the increased damage observed in MG dropout, MGD, and reduced corneal sensitivity. However, we could not identify a correlation between MG dropout and corneal sensitivity. The current study agrees with what has been reported by other authors, that MGD parameters such as MG expressibility, meibum quality, and Marx’s line were more severely affected in patients with DM than in patients with no diabetes [12,13,15]. In patients who are going to undergo cataract surgery, it is important to look for DED before and after surgery in order to establish appropriate treatments.

This study observed significant correlations between MG dropout and TBUT, MG expressibility, and Marx’s line in patients with DM with NPDR. Similar results were reported by Yang Qin et al., who also found a negative correlation between MG dropout and the first non-invasive breakup time (NIBUT) but did not find any correlation with any other DED or MGD parameter [12]. The absence of a correlation between most of the dry eye parameters is an expected finding that has been previously reported. This is due, among other factors, to the fact that dry eye pathogenesis is multifactorial and has multiple etiologies; therefore, the dry eye parameters will frequently be different between patients [38,39]. Tear osmolarity was normal in patients with NPDR and in patients with no diabetes. MMP-9 was positive in less than 5% of the patients in both groups. High tear osmolarity and positive tear MMP-9 are complementary diagnostic DED tests that can be of use in identifying the inflammatory aspect of DED, but these are not the gold standard and are not stand-alone DED diagnostic tests [1,2,3]. MMP-9 and tear osmolarity could be elevated in patients with inflammatory types of DED, such as Sjögren syndrome or graft versus host disease DED [1,3]. This study population of patients with DM listed for cataract surgery were not patients from a dry eye clinic and did not have previous diagnosis or dry eye; in addition, they had good glycemic control in preparation for cataract surgery. Therefore, it can be expected that our study population did not have an inflammatory ocular surface disease, and therefore, MMP-9 and tear osmolarity could be expected to be within normal limits. Additionally, the effect of DM on DED is more because of autonomic dysregulation and neuropathic changes, and not of an inflammatory cause [2,6,7,8]. Lastly, due to the multifactorial characteristics of DED, it is expected that not all the evaluated dry eye parameters were abnormal.

The limitations of the present study are that the use of artificial tears and previous diagnosis of DED were not recorded, and a control group of patients with diabetes without NPDR was not included. Another limitation was that fast blood glycemic and glycosylated hemoglobin A1c (HbA1c) were not evaluated. However, we believe that, by focusing on patients with NPDR regardless of their blood sugar level, we have a uniform population of patients for whom diabetes has already caused complications in their eyes. Therefore, the study population included only patients with NPDR with a long duration of DM and not a mixed population of patients with diabetes with or without diabetic retinopathy and different diabetes durations. The strengths of this study include an objective evaluation of MG dropout (ImageJ software), analysis of the meiboscore grade, and the actual percentage of MG dropout. This is relevant since most of the previously published studies only performed a subjective meiboscore evaluation of MG dropout and not an objective evaluation and did not mention numerical percentages of MG dropout. In addition, this was a case–control study matched for age and gender; a comprehensive evaluation of DED and MGD was performed; and correlations with age, gender, diabetes duration, and other DED and MGD parameters were performed.

## 5. Conclusions

This study shows that patients with diabetes with NPDR have a greater MG dropout percentage and a greater meiboscore grade than patients with no diabetes. All patients showed some degree of MG dropout. Age showed a weak positive correlation with MG dropout, but the duration of diabetes and gender were not correlated with MG dropout. The percentage of MG dropout negatively correlated with TBUT and MG expressibility in patients with DM. More severe MGD and DED parameters were observed in patients with DM with NPDR than in patients with no diabetes. It is important to recognize that these patients present more serious alterations in their ocular surface, and for this reason, it is important to make an early diagnosis in order to initiate timely treatments. Although the meiboscore grade classification is very helpful and widely used, it is important to evaluate meibography objectively and achieve a numerical percentage of MG dropout to allow for the detection of more subtle changes over time [40]. Future studies evaluating the impact that different degrees of diabetic retinopathy might have on the magnitude of MG dropout will be of interest, and the impact that cataract surgery has on DED in the diabetic population will further improve the knowledge on the impact of diabetic complications at the ocular surface.

## Figures and Tables

**Table 1:** Selected studies about meibomian gland dropout on type 2 diabetes.

Author/Country/Year	N= (Eyes)	MG Dropout Assessment on Meibography	Meiboscore DM vs. NDM	MG Dropout DM vs. NDM	Age and DM Duration (Years)	MG Dropout Correlation with Age and DM Duration	Other MGD Findings in DM vs. NDM
Mohamed-Noriega et al. Mexico, 2024. (This study)	DM: 98 (all NPDR) NDM: 106	Objective, with ImageJ and meiboscore	Higher in DM 3.8 ± 0.8 vs. 3.4 ± 1.0, *p* = 0.001	Greater in DM 45 ± 11 vs. 39 ± 13, *p* = 0.016	Age: 67 ± 10 Duration: 18 ± 9	Correlation with age (r = 0.178, *p* = 0.014). No correlation with DM duration	Worse MG expressibility (3. 9 ± 1.6 vs. 4.4 ± 2.1, *p* = 0.017), meibum quality (1.9 ± 0.8 vs. 1.7 ± 0.5; *p* = 0.019), and Marx’s line (1.6 ± 0.8 vs. 1.8 ± 0.5, *p* < 0.001)
Yang Q et al. China, 2023 [12]	DM + DED: 30 NDM no DED: 16	Objective, with ImageJ and meiboscore		Greater in DM 33.5 ± 8.2 vs. 16.5 ± 6.6, *p* = 0.001	Age: 65 ± 9 Duration: 12 ± 8	Correlation with DM duration (r = 0.509, *p* < 0.05) Age: not analyzed	Worse meibum score (2.2 ± 0.6 vs. 0.7 ± 0.6, *p* = 0.003)
Wu, Huping, et al. China, 2022 [13]	DM: 99 NDM: 40	Subjective, with meiboscore No ImageJ	Higher in DM 3.5 ± 1.0 vs. 2.3 ± 0.8, *p* < 0.001		Age: 60 ± 6 Duration: 5 ± 3	Correlation with age (β = 0.362, *p* = 0.001) and DM duration (*p* < 0.001)	Worse meibum score (25.0 ± 6.1 vs. 14.5 ± 4.1, *p* < 0.001)
Yu T et al. China, 2019 [14]	DM: 132 NDM: 100	Subjective, with meiboscore No ImageJ	Higher in DM (Z = −4.057, *p* < 0.001)		Age: 60 ± 8 Duration: 8 ± 5	Correlation with DM duration (r = 0.509, *p* < 0.05) Age: not analyzed	
Lin X et al. China, 2017 [15]	DM: 78 NDM: 108	Subjective, with meiboscore No ImageJ	Higher in DM (4.3 ± 1.4 vs. 3.6 ± 1.4, *p* = 0.004)		Age: 67 ± 2 Duration: 9 ± 5	Not analyzed	Worse MG expressibility (*p* = 0.039) and lid margin abnormality score (*p* = 0.04)
Yu T et al. China, 2016 [6]	DM: 118 NDM: 100	Subjective, with meiboscore No ImageJ		More prevalence of MG dropout in DM: 57% vs. 33%, *p* ≤ 0.001	Age: 60 ± 8 Duration: No data	Not analyzed	

DM: diabetes mellitus; NDM: non diabetes; MG: meibomian gland; MGD: meibomian gland dysfunction; NPDR: non-proliferative diabetic retinopathy; DED: dry eye disease.

**Table 2:** Demographics, dry eye, and meibomian gland disfunction evaluations.

Variable	Patients with No Diabetes *n* = 106 Eyes	Patients with Diabetes with NPDR *n* = 98 Eyes	*p*
Men	26 (49.1%)	24 (48.9%)	0.896
Women	27 (50.9%)	25 (51.1%)	
Age	66.7 ± 9	67.1 ± 10	0.982
Duration of diabetes	N/A	18 ± 9 (3–31)	N/A
OSDI	38.9 ± 20	45.98 ± 22	0.535
TBUT	7.0 ± 2.8	6.7 ± 2.8	0.969
Osmolarity	303 ± 19	298 ± 17	0.230
MMP-9 positive	5 (4.7%)	3 (3.0%)	0.725
Oxford staining grade	0.8 ± 1.1	1.4 ± 1.2	**0.001**
NEI staining grade	0.9 ± 1.1	1.7 ± 1.3	**0.001**
Schirmer without anesthesia	14.7 ± 8.0	17.0 ± 9.1	0.115
Schirmer with anesthesia	13.3 ± 6.2	14.4 ± 8.0	0.120
Corneal esthesiometry	5.9 ± 0.4	5.4 ± 1.1	**<0.001**
MG expressibility	4.4 ± 2.1	3.9 ± 1.6	**0.017**
Meibum quality	1.7 ± 0.5	1.9 ± 0.8	**0.019**
Marx’s line	1.6 ± 0.8	1.8 ± 0.5	**<0.001**

NPDR: non-proliferative diabetic retinopathy; OSDI: Ocular Surface Disease Index; TBUT: tear breakup time; MG: meibomian gland; N/A: not applicable. Pearson’s X^2^ or independent samples *t*-test; significance at *p* < 0.05. Bold means statistically significant.

**Table 3:** Meibomian gland (MG) dropout and meiboscore.

	Patients with No Diabetes Mean ± SD (min–max)	Patients with Diabetes with NPDR Mean ± SD (min–max)	*p*
Superior eyelid			
Arita’s grade (0–3)	1.4 ± 0.6 (1–3)	1.8 ± 0.5 (1–3)	**<0.001**
MG dropout (%)	34.6 ± 13.9 (7–71)	42.3 ± 13.6 (13–72)	**<0.001**
Inferior eyelid			
Arita’s grade (0–3)	1.7 ± 0.6 (1–3)	2.0 ± 0.5 (1–3)	**<0.001**
MG dropout (%)	43.0 ± 17.3 (6–81)	50.8 ± 14.4 (8–75)	**0.001**
Superior vs. inferior eyelid			
*p*-Value Arita’s grade	**<0.001**	**0.014**	N/A
*p*-value MG dropout	**<0.001**	**<0.001**	N/A
Both eyelids			
Meiboscore grade * (0–6)	3.4 ± 1.0 (2–6)	3.8 ± 0.8 (2–5)	**0.002**
MG dropout (%)	39.0 ± 13.2 (15–72)	45.1 ± 10.5 (16–64)	**<0.001**

NPDR: non-proliferative diabetic retinopathy; SD: standard deviation; N/A: not applicable; *t*-test, significance at *p* < 0.05. Bold means statistically significant. * Arita’s meiboscore.

**Table 4:** Total meiboscore grade (n, %).

Meiboscore Grade	Patients with No Diabetes	Patients with Diabetes with NPDR	*p*
0	0 (0)	0 (0)	**0.016**
1	0 (0)	0 (0)	
2	20 (19)	4 (4)	
3	39 (37)	22 (24)	
4	34 (32)	49 (55)	
5	7 (6)	13 (14)	
6	5 (4)	1 (1)	

NPDR: non-proliferative diabetic retinopathy. Pearson’s X^2^, significance at *p* < 0.05. Bold means statistically significative.

**Table 5:** Correlations between the percentage of MG dropout and dry eye parameters.

Variable	Patients with No Diabetes		Patients with Diabetics with NPDR	
	r	*p*	r	*p*
OSDI	−0.013	0.895	0.063	0.560
TBUT	0.147	0.147	−0.295	**0.006**
Osmolarity	0.110	0.281	−0.095	0.385
Oxford staining grade	−0.061	0.548	0.069	0.519
NEI staining grade	0.001	0.954	−0.037	0.731
Schirmer without anesthesia	0.028	0.784	−0.067	0.542
Schirmer with anesthesia	0.129	0.203	−0.003	0.984
Corneal esthesiometry	0.046	0.650	0.130	0.563
Marx’s line	−0.087	0.391	0.371	**0.007**
MG expressibility	−0.105	0.300	−0.428	**0.002**
Meibum quality	0.029	0.775	0.307	0.730

MG: meibomian gland; OSDI: Ocular Surface Disease Index; TBUT: tear breakup time; NPDR: non-proliferative diabetic retinopathy; r: correlation coefficient. Pearson bilateral rank correlation test. Significance at *p* < 0.05. Bold means statistically significant.

## Data Availability

The database that supports these findings is available upon request via e-mail to the corresponding author.
